# Supplementary material for: Complex‐centric proteome profiling by SEC‐SWATH‐MS
Source: Mol Syst Biol. 2019 Jan 14;15(1):e8438. doi: 10.15252/msb.20188438 (PMC6346213; doi:10.15252/msb.20188438)
Supplement: Supplementary file 8 — Dataset EV7 [file MSB-15-e8438-s008.zip › feature_plots_string/P26232.pdf]

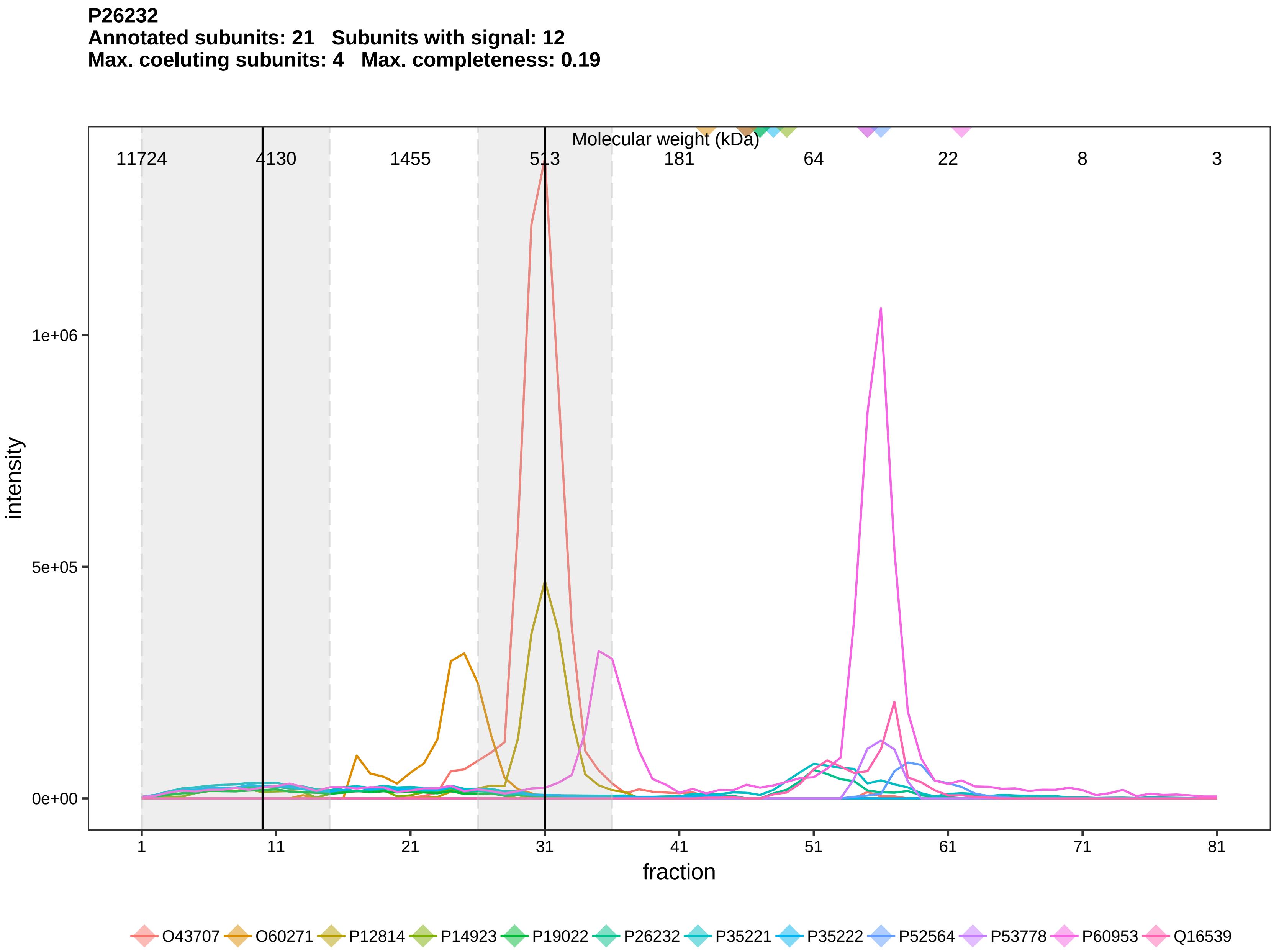
P26232
Annotated subunits: 21 Subunits with signal: 12
Max. coeluting subunits: 4 Max. completeness: 0.19
Molecular weight (kDa)
11724
4130
1455
513
181
64
22
8
3
intensity
1e+06
5e+05
0e+00
1
11
21
31
41
51
61
71
81
fraction
O43707
O60271
P12814
P14923
P19022
P26232
P35221
P35222
P52564
P53778
P60953
Q16539
